# Mortality risk after the first occurrence of osteoporotic vertebral compression fractures in the general population: A nationwide cohort study

**DOI:** 10.1371/journal.pone.0291561

**Published:** 2023-09-14

**Authors:** Hee Jung Son, Se-Jun Park, Jeong-Keun Kim, Jin-Sung Park

**Affiliations:** 1 Department of Orthopedic Surgery, Nowon Eulji Medical Center, Eulji University School of Medicine, Seoul, South Korea; 2 Department of Orthopedic Surgery, Spine Center, Samsung Medical Center, Sungkyunkwan University School of Medicine, Seoul, South Korea; Taipei Medical University, TAIWAN

## Abstract

Osteoporotic vertebral compression fractures (OVCF) can cause severe pain, changes in balance, gait velocity, muscle fatigue, risk of falls, and subsequent fractures. Thus, OVCF significantly lowers the individual’s health-related quality of life. Additionally, OVCF may increase patient mortality rates. However, studies on post-OVCF mortality are limited. This study aimed to evaluate mortality risk after the first occurrence of OVCF in the general population using a nationwide dataset from the Korean National Health Insurance System. We identified 291,203 newly diagnosed patients with OVCF and 873,609 patients without OVCF at a ratio of 1:3 matched by sex and age between 2010 and 2012. We investigated the latent characteristics of patients’ demographic information and chronic comorbidities that could affect mortality when diagnosed with OVCF. By comparing the cohort data, the hazard ratio for subsequent mortality in patients with OVCF was calculated and adjusted based on several risk factors. Despite adjusting for demographic characteristics and chronic comorbidities, the risk of mortality was 1.22 times higher in the OVCF cohort than in the control group. Multivariate analysis showed that male sex, old age, low-income status, and high Charlson Comorbidity Index were associated with a higher risk of mortality. In addition, the presence of chronic comorbidities, including diabetes mellitus, ischemic heart disease, stroke, chronic obstructive pulmonary disease, cancer, and end-stage renal disease, was shown to increase the risk of mortality. This population-based cohort study showed that newly diagnosed OVCF significantly increased the subsequent risk of mortality. Moreover, post-OVCF mortality is influenced by demographic characteristics and chronic comorbidities.

## Introduction

As the aging population increases worldwide, the incidence of osteoporosis and osteoporotic fractures, such as spine, hip, wrist, and shoulder fractures, also increases. Among fragility fractures related to osteoporosis, osteoporotic vertebral compression fractures (OVCF) are the most common (almost 50%) and tend to occur earlier than other major osteoporotic fractures, such as hip fractures [1–4].

There are approximately 1,416,000 cases of OVCF worldwide each year, and approximately 40% of women experience OVCF at least once during their lifetime [5]. A global OVCF study found that the age-standardized OVCF incidence was higher in the United States and Asia than in Europe [6]. In Korea, OVCF patients with OVCF increased from 117,361 in 2012 to 139,889 in 2016 and the 5-year incidence of OVCF per 100,000 persons was 852.24 cases [7]. As the number of vertebral fractures in Korea is expected to increase in the near future, the socioeconomic burden of vertebral fractures will also increase [8].

OVCF can cause severe pain as well as changes in balance, gait velocity, muscle fatigue, risk of falls, and subsequent fractures. Thus, OVCF significantly lowers health-related quality of life [9–11]. In addition, OVCF may increase patient mortality due to kyphosis-induced crowding of internal organs, pulmonary disease, and deep vein thrombosis. Mortality after OVCF is determined by the severity of the vertebral fracture, outcome of the intervention (vertebroplasty or kyphoplasty), complications, and demographics [12, 13]. Thus, direct causal mortality after OVCF is difficult to determine and has not been well studied. However, epidemiological trends can be determined using a nationwide sample size. This study aimed to evaluate the mortality risk after the first occurrence of OVCF in the general population.

## Materials and methods

### Data source

This study analyzed data from the Korean National Health Insurance System (KNHIS) between 2010 and 2012. In South Korea, all citizens are covered by the National Health Insurance (NHI) system except for those undergoing cosmetic surgery and treatment due to traffic or industrial accidents. It covers 97% of the population, and the remaining 3% of the lowest-income households are covered with Medical Aid Program. The database included diagnoses classified according to the International Classification of Diseases, 10^th^ revision (ICD-10), demographic characteristics, inpatient and outpatient care, prescriptions, and procedures for almost all patients in South Korea.

### Study design and cohort

OVCF is defined as a vertebral fracture due to low-level (or low-energy) trauma, which is a force equivalent to falling from a standing height or less [14]. To obtain a more accurate study population of OVCF, we developed algorithms for patients aged ≥60 years rather than simply using diagnostic codes. First, osteoporotic vertebral fracture codes (ICD-10, M8008-6, M8088-6, and M8098-6) were considered as osteoporosis-related fragility fractures. Second, vertebral fracture codes (ICD-10, S220, S221, S320) with osteoporosis codes (ICD-10, M810, M818, M819), and without procedure codes, such as posterior fixation surgery within one month (ICD-10, N0468, N0469, N1460, N1469), were used to exclude high-energy fractures. OVCF is defined when the first or second criteria was met. Patients with a history of OVCF were excluded.

**Fig 1:**
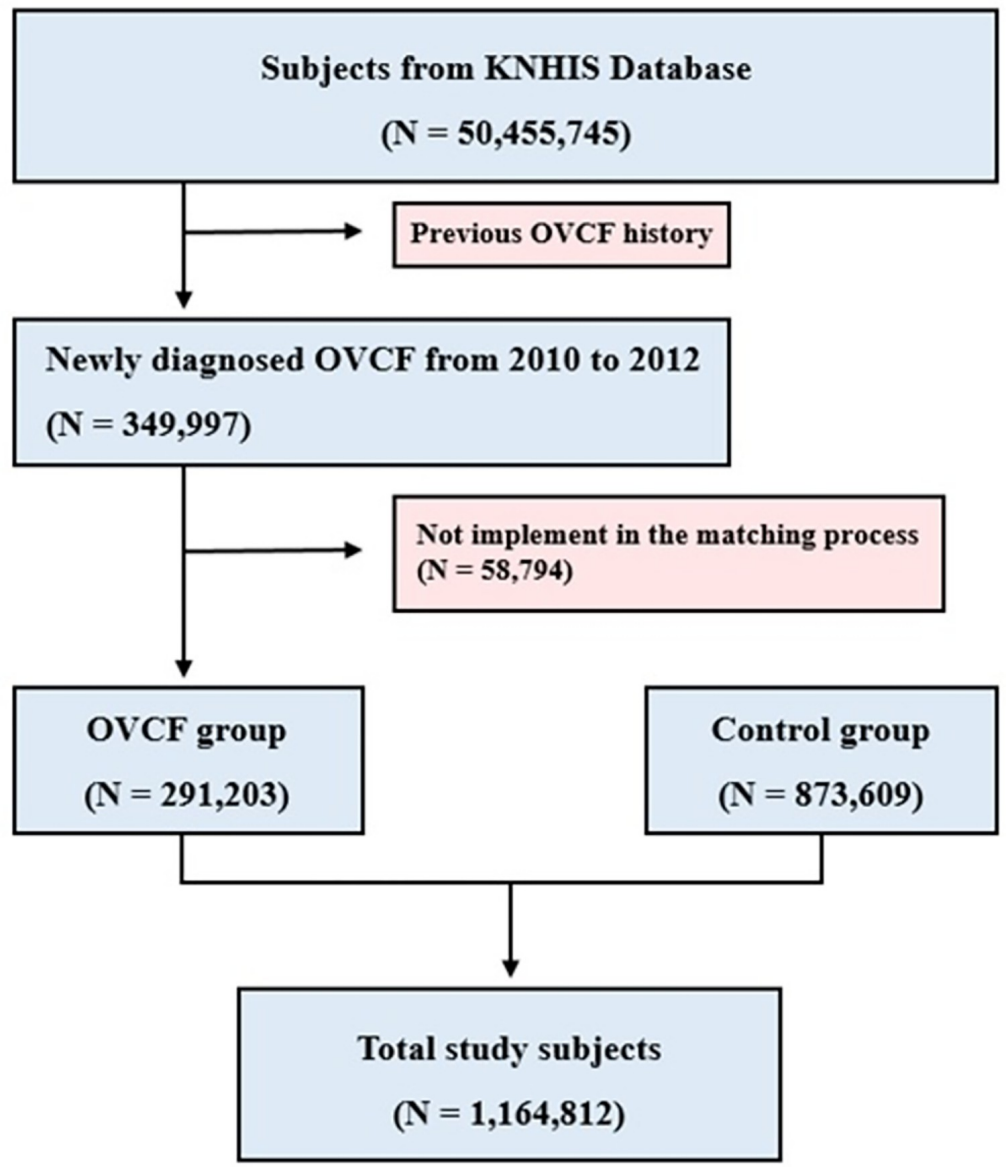
Flowchart of the subjects from the Korean National Health Insurance System.

Sex and age were matched in a 1:3 ratio for newly diagnosed OVCF. To minimize selection bias, the patients in the control group were randomized before selection. The group without three control individuals was excluded from the study (n = 58,794). Finally, we compared the mortality rate of patients newly diagnosed with OVCF (n = 291,203) with that of patients in the control group (n = 873,609) (Fig 1). Mean follow-up period was 5.87 years.

The demographic characteristics and chronic comorbidities were investigated after matching the OVCF and control groups. Low-income status was defined as <20% of the general population, and urban residence was defined as a city with a population of one million or more (Seoul, Busan, Incheon, Daegu, Daejeon, Ulsan, and Gwangju).

The comorbidities, selected with ICD-10 codes, included diabetes mellitus (ICD-10, E11-14), hypertension (ICD-10, I10-13, 15), ischemic heart disease (ICD-10, I20-25), stroke (ICD-10, I63, 64), congestive heart failure (ICD-10, I50), chronic obstructive pulmonary disease (ICD-10, J44), cancer (ICD-10 codes beginning with “C”), and end-stage renal disease (ICD-10, N18, 19). Disease was confirmed when the code was present in subjects who underwent three or more visits for principal and/or secondary diagnosis. To increase the validity of the diagnostic codes, the suitability of the prescription drugs for the selected diseases was reviewed. In addition, the Charlson Comorbidity Index (CCI) was used to evaluate the health status of patients precisely [15]. A subgroup analysis of the mortality rate in patients with OVCFs according to demographic characteristics and comorbidities was also performed.

### Ethical statement

This study was approved by the institutional review board of Nowon Eulji Medical Center (IRB approval no.2023-02-002). The need for informed consent was waived by the board because of the retrospective nature of this study.

### Statistical analysis

The Chi-square test was used to evaluate demographic characteristics and CCI. Student t-test was used to analyze mean CCI. The cumulative mortality rate was analyzed using the Kaplan–Meier method. Cox proportional hazards regression analysis was used to identify the hazard ratio (HR) and 95% confidence interval (CI) of mortality in patients with OVCF compared to the control group. A multivariate Cox proportional hazards model was used to verify the risk factors for mortality and the adjusted HRs (Model 1: age and sex; Model 2: age, sex, income, residence, and comorbidity). SAS version 9.3 (SAS Institute Inc., Cary, NC, USA) was used for statistical analysis. Statistical significance was set at p < 0.05.

## Results

### Demographic characteristics

The demographic characteristics and chronic comorbidities of the OVCF and control groups are presented in Table 1. The sex and age proportions were identical in both groups because they were matched in a 1:3 ratio. Patients with OVCF generally had a low-income status and lived in rural areas (p < 0.001). Comorbidities and CCI scores were significantly higher in the OVCF group than in the control group (p < 0.001).

**Table 1:** Comparison of demographic and comorbidities.

	Patients with OVCF (n = 291,203)	Controls (n = 873,609)	p
Sex (male)	44,808 (15.4%)	134,424 (15.4%)	1.000
Age			1.000
≥ 60–69 years	87,241 (30.0%)	261,723 (30.0%)	
≥ 70–79 years	136,625 (46.9%)	409,875 (46.9%)	
≥ 80–89 years	60,966 (20.9%)	182,898 (20.9%)	
≥ 90 years	6,371 (2.2%)	19,113 (2.2%)	
Low-income status (low 20%)	85,128 (29.2%)	236,165 (27.0%)	< 0.001*
Residence (urban)	103,709 (35.6%)	347,169 (39.7%)	< 0.001*
CCI			< 0.001*
0	43,972 (15.1%)	242,333 (27.7%)	
1–2	119,907 (41.2%)	371,105 (42.5%)	
3–5	95,076 (32.7%)	212,257 (24.3%)	
≥ 6	32,248 (11.1%)	47,914 (5.5%)	
Mean CCI	1.89 ± 1.91	2.64 ± 2.21	< 0.001*

Values are given as mean ± standard deviation.

*Significant difference

OVCF, osteoporotic vertebral compression fractures; CCI, Charlson Comorbidity Index.

### Risk of mortality

**Fig 2:**
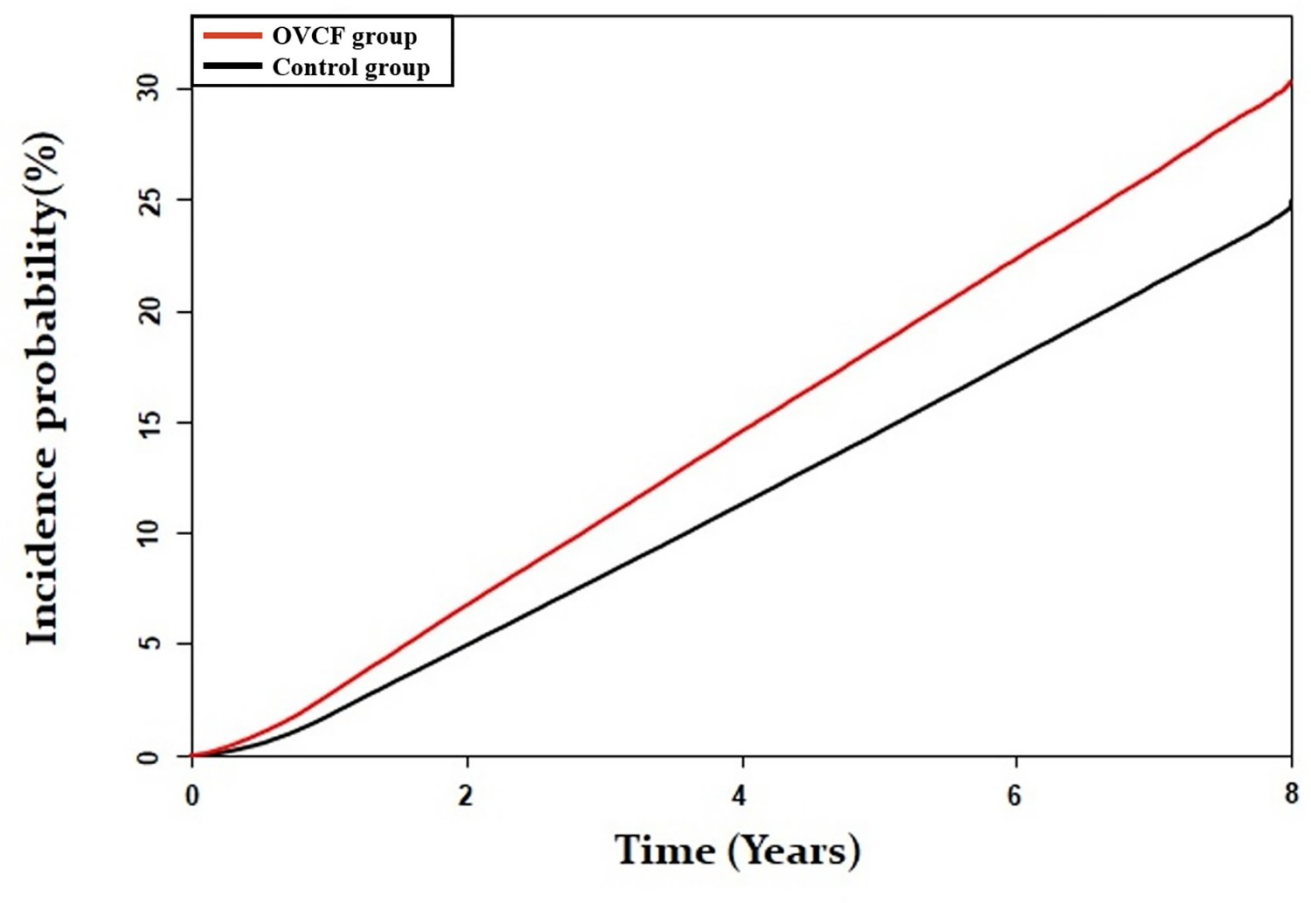
The Kaplan–Meier curves showed significantly higher cumulative rate of mortality of osteoporotic vertebral compression fractures.

In the OVCF and control groups, 24.3% (70,752/291,203) and 19.6% (170,806/873,609) of the patients died, respectively, during follow-up. There was a higher risk of mortality in OVCF patients than in the control group (crude HR:1.28, 95% CI:1.27–1.30; and adjusted HR:1.22, 95% CI:1.20–1.23) (Table 2). The Kaplan–Meier curves showed a significantly higher cumulative mortality rate in the OVCF group than in the control group (p < 0.001). Additionally, the interval between the two curves in the OVCF and control groups increased over time (Fig 2).

**Table 2:** Risk of mortality in patients with OVCF.

Patient group	N	Death	Duration	Rate	HR (95% CI)	
					Crude HR	Adjusted HR
Controls	873,609	170,806	5161842.0	33.1	1 (reference)	1 (reference)
Patients with OVCF	291,203	70,752	1670414.2	42.36	1.28 (1.27–1.30)	1.22 (1.20–1.23)

Crude HR; age, sex.

Adjusted HR; age, sex, income, residence, comorbidity.

OVCF, osteoporotic vertebral compression fractures; HR, hazard ratio.

### Subgroup analysis of the risk of mortality in patients with OVCF

Patients with OVCF with male sex, old age, low-income status, and high CCI were associated with a higher risk of mortality. There was little difference in the mortality risk based on residence (Table 3). Additionally, the presence of chronic comorbidities increases the risk of mortality. Patients with OVCF with congestive heart failure have the highest risk (HR:2.26, 95% CI:2.21–2.30), followed by those with cancer (HR:2.09, 95% CI:2.04–2.14) and with stroke (HR:2.07, 95% CI:2.02–2.12) (Table 4).

**Table 3:** Subgroup analysis of the rate of mortality in patients with OVCF.

Variables		Crude HR (95% CI)	Adjusted HR
Sex	Male	1.921 (1.903–1.939)	1.801 (1.784–1.818)
	Female	1 (reference)	1 (reference)
Age group	60–64 years	1 (reference)	1 (reference)
	65–69 years	1.701 (1.65–1.754)	1.63 (1.58–1.681)
	70–74 years	3.204 (3.115–3.296)	2.927 (2.845–3.01)
	75–79 years	6.122 (5.956–6.292)	5.467 (5.318–5.619)
	80–84 years	11.536 (11.225–11.856)	10.35 (10.07–10.638)
	85–89 years	20.245 (19.688–20.818)	18.893 (18.373–19.428)
	≥ 90 years	32.77 (31.803–33.765)	32.319 (31.365–33.302)
Residence	Urban	1 (reference)	1 (reference)
	Rural	1.141 (1.132–1.151)	1.029 (1.021–1.038)
Incomes	Other	1 (reference)	1 (reference)
	Low 20%	1.341 (1.329–1.352)	1.205 (1.194–1.215)
Comorbidity	0	1 (reference)	1 (reference)
	1–2	1.316 (1.301–1.331)	1.3 (1.285–1.316)
	3–5	1.962 (1.939–1.986)	1.87 (1.848–1.892)
	≥ 6	3.467 (3.416–3.518)	3.156 (3.11–3.203)

Crude HR; age, sex.

Adjusted HR; age, sex, income, residence, comorbidity.

OVCF, osteoporotic vertebral compression fractures; HR, hazard ratio

**Table 4:** The rate of mortality according to comorbidity in patients with OVCF.

Variables		Crude HR (95% CI)	Adjusted HR
DM	No	1 (reference)	1 (reference)
	Yes	1.31 (1.30–1.32)	1.46 (1.44–1.47)
HTN	No	1 (reference)	1 (reference)
	Yes	1.17 (1.16–1.18)	1.00 (0.99–1.01)
IHD	No	1 (reference)	1 (reference)
	Yes	2.74 (2.66–2.83)	1.58 (1.53–1.63)
Stroke	No	1 (reference)	1 (reference)
	Yes	3.09 (3.02–3.17)	2.07 (2.02–2.12)
CHF	No	1 (reference)	1 (reference)
	Yes	4.20 (4.12–4.29)	2.26 (2.21–2.30)
COPD	No	1 (reference)	1 (reference)
	Yes	1.60 (1.59–1.62)	1.28 (1.27–1.29)
Cancer	No	1 (reference)	1 (reference)
	Yes	2.2 (2.17–2.23)	2.09 (2.04–2.14)
ESRD	No	1 (reference)	1 (reference)
	Yes	2.46 (2.40–2.53)	1.90 (1.84–1.96)

Crude HR; age, sex.

Adjusted HR; age, sex, income, residence, comorbidity.

OVCF, osteoporotic vertebral compression fracture; HR, hazard ratio; DM, diabetes mellitus; HTN, hypertension; IHD, ischemic heart disease; CHF, congestive heart failure; COPD, chronic obstructive pulmonary disease; ESRD, end-stage renal disease.

## Discussion

Numerous studies have reported increased mortality rates after OVCF. In a study using Medicare claims in the United States, even after controlling for the effects of comorbidities, the mortality rate of patients with vertebral fractures was approximately twice that of matched controls, and was higher in men than in women [16]. Kim et al. [8] analyzed data from the KNHIS database and reported that the cumulative mortality rate in the first year after vertebral fractures decreased from 8.51% (5,955/69,972) in 2008 to 7.0% (7,187/102,642) in 2012. In addition, the mortality rate after OVCF was higher in more severe and multiple fractures, was highest immediately following the fracture, and decreased with time [17, 18].

It is uncertain whether the link between OVCF and the mortality rate is causal because deaths are primarily due to comorbidities. In other words, high mortality after OVCF may be a result of poor general health and physical function because patients with osteoporotic fractures typically have poor general health, physical function, and many comorbidities [19]. However, even patients with OVCFs without comorbidities have been reported to have a higher mortality rate than the matched controls [16]. OVCF itself may be attributed to an increase in mortality rate, either directly or indirectly. Although it is difficult to explain exactly why the mortality rate of patients with OVCF is higher than that of the controls, it is supposed that OVCF causes a prolonged bedridden state, hospital stays, and kyphosis resulting in pulmonary disease, deep vein thrombosis, and exacerbated comorbidities [8, 19].

Mortality and morbidity are associated with osteoporotic fractures. Hip fractures are associated with a higher mortality rate than vertebral fractures; however, the opposite is true for morbidity [14]. Because OVCF tends to occur before other major osteoporotic fractures, a newly diagnosed OVCF may be a sentinel sign of future subsequent osteoporotic fractures, progressive functional decline, aggravation of comorbidities, and higher mortality [11, 19, 20]. According to a cohort study using the national claims database by Yoo et al. [11], during a median follow-up period of 3 years, the incidence of subsequent OVCF was 15.5%, and the mortality rate of subsequent fracture patients was 1.2 times than that of non-subsequent fracture patients. In this study, we found that first OVCF significantly increased the risk of mortality.

Despite the importance of osteoporosis treatment, it has not been implemented as expected. Barton et al. [21] reported that 73% of patients with first incidence of OVCF did not receive antiresorptive therapy before or after the fracture. In a retrospective study by Iida et al. [22], bisphosphonate treatment for osteoporosis was beneficial for fracture prevention and mortality after OVCF. Interventions for OVCF, such as vertebroplasty or kyphoplasty, are effective not only for pain relief but also for reducing morbidity and mortality. In a meta-analysis of more than 2 million patients, patients undergoing cement augmentation were 22% less likely to die within 10 years than those who received conservative treatment [13]. Therefore, the prevention of OVCF through osteoporosis treatment and aggressive treatment of OVCF itself are important to reduce the post-OVCF mortality rate.

According to a nationwide epidemiological study by Park et al. [1], the prevalence and incidence of osteoporosis and OVCF were higher in the Medical Aid group than in the NHI group. In other words, a low income may be related to osteoporosis and OVCF. Moreover, we found that low-income status was associated with a higher risk of mortality. Thus, a nationwide fracture-prevention program involving osteoporosis treatment for all populations, including low-income households, is essential.

This study has several limitations. First, it was difficult to distinguish between OVCF and vertebral fractures caused by high-energy trauma using the KNHIS data. However, we used algorithms that included procedure codes for patients aged ≥60 years rather than simply using diagnostic codes to distinguish OVCF, which ensured that included cases were due to low-energy trauma as far as possible. Second, considering asymptomatic OVCF, mortality may be underestimated because only one-third of OVCF cases are clinically diagnosed and an even smaller proportion of patients visit the hospital [19]. Third, the bone mineral density (BMD) score could not be estimated because the KNHIS data did not include BMD score, only diagnosis by ICD-10 codes. Therefore, it was not possible to directly compare BMD score between the two groups. Finally, the ICD-10 codes may have been incorrectly entered. To maximize reliability, we confirmed the diagnostic code when the code was present in subjects who underwent three or more visits, and reviewed the suitability of prescription drugs for selected diseases. Despite these limitations, this study had several strengths, including the analysis of a very large data set from the KNHIS, establishment of age- and sex-matched controls, and adjustment for chronic comorbidity variables.

## Conclusions

This population-based cohort study showed that newly diagnosed OVCF significantly increased the subsequent risk of mortality. Moreover, post-OVCF mortality is influenced by demographic characteristics and chronic comorbidities.

## Supporting information

S1 Checklist STROBE statement—checklist of items that should be included in reports of observational studies. (DOCX)
